# KIF24 depletion induces clustering of supernumerary centrosomes in PDAC cells

**DOI:** 10.26508/lsa.202201470

**Published:** 2022-07-08

**Authors:** Yu Mashima, Hayato Nohira, Hiroki Sugihara, Brian David Dynlacht, Tetsuo Kobayashi, Hiroshi Itoh

**Affiliations:** 1 Division of Biological Science, Graduate School of Science and Technology, Nara Institute of Science and Technology, Ikoma, Japan; 2 Department of Pathology and Cancer Institute, Smilow Research Center, New York University School of Medicine, New York, NY, USA

## Abstract

Clustering of supernumerary centrosomes, which potentially leads to cell survival and chromosomal instability, is frequently observed in cancers. However, the molecular mechanisms that control centrosome clustering remain largely unknown. The centrosomal kinesin KIF24 was previously shown to restrain the assembly of primary cilia in mammalian cells. Here, we revealed that KIF24 depletion suppresses multipolar spindle formation by clustering centrosomes in pancreatic ductal adenocarcinoma (PDAC) cells harboring supernumerary centrosomes. KIF24 depletion also induced hyper-proliferation and improved mitotic progression in PDAC cells. In contrast, disruption of primary cilia failed to affect the proliferation and spindle formation in KIF24-depleted cells. These results suggest a novel role for KIF24 in suppressing centrosome clustering independent of primary ciliation in centrosome-amplified PDAC cells.

## Abstract (precis)

Depletion of the centrosomal kinesin KIF24, known to restrain the assembly of primary cilia, suppresses multipolar spindle formation by clustering centrosomes in centrosome-amplified PDAC cells.

## Graphical Abstract

**Figure:**
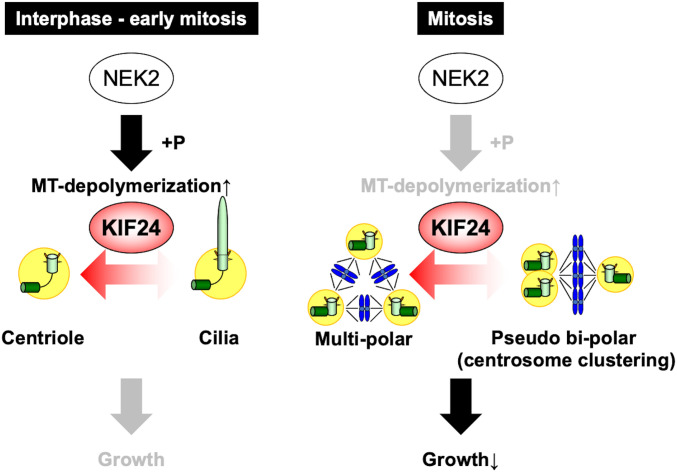

## Introduction

The centrosome comprises centrioles and a pericentriolar matrix (PCM). The two cylinder-like centrioles in the G1 phase are duplicated through the S–G2 phase, and the two-paired centrioles ensure bipolar spindle formation during mitosis. As new centrioles are duplicated from the older centriole, the differentially aged centrioles in the G1 phase are termed the older mother and the younger daughter centrioles. The mother centriole, characterized by the distal and sub-distal appendages, nucleates the primary cilium during the interphase, generally in the G0 phase. This single hair-like organelle is ubiquitously expressed in mammalian cells containing specific receptors and channels to receive multiple signaling molecules. Given that both the spindle and primary cilium share the centriole for their assembly, they are thought to be incompatible with each other in normal somatic cells (Kobayashi & Dynlacht, 2011; Sánchez & Dynlacht, 2016).

Contrary to normal cells, the numbers of centrosomes and primary cilia are anomalous in many cancer types. The number of primary cilia decreases in various cancers, which probably causes aberrant signal transduction and cell cycle progression (Eguether & Hahne, 2018; Liu et al, 2018). Supernumerary centrosomes are also observed in cancers that potentially nucleate multipolar spindles during mitosis, thereby leading to cell death. Despite this, the cancer cells can often avoid the detrimental multipolar spindles by forming pseudo bipolar spindles in which the multiple centrosomes are clustered into one spindle to allow bipolar separation, a process termed centrosome clustering (Funk et al, 2016; Milunović-Jevtić et al, 2016; Levine & Holland, 2018). Pseudo bipolar mitosis not only allows completion of cell division and survival of the daughter cells but also occasionally induces certain tumor cell hallmarks, such as chromosome segregation errors, aneuploidies, and invasion (Ganem et al, 2009).

Pancreatic ductal adenocarcinoma (PDAC) accounts for over 90% of pancreatic cancers, with a 5-yr survival rate of less than 10% (Adamska et al, 2017). Similar to other cancers, primary cilia are decreased in PDAC lesions and cell lines (Seeley et al, 2009; Schimmack et al, 2016). Most PDAC samples harbor actively mutated oncogenic KRAS, and KRAS signaling is known to inhibit primary ciliogenesis in PDAC (Seeley et al, 2009). We have previously revealed that KRAS and a histone deacetylase HDAC2 suppress primary ciliation by regulating Aurora A kinase (AurA) expression in PDAC cells (Kobayashi et al, 2017; Kobayashi & Itoh, 2017). Furthermore, the suppression of ciliogenesis has been reported to promote the transformation of normal pancreatic ductal cells into cancer cells (Deng et al, 2018). Centrosomal amplification and clustering also occur in PDAC (Sato et al, 1999; Zhu et al, 2005; Mittal et al, 2015; Ansari et al, 2018); however, the molecular mechanisms underlying the centrosome clustering remain largely unclear.

Kinesin family member 24 (KIF24) belongs to the Kinesin-13 subfamily with a kinesin domain in its middle region (Miki et al, 2005). KIF24 localizes to centrioles and interacts with two centriolar proteins, CP110 and CEP97, which prevent the assembly of the primary cilia in cycling mammalian cells (Kobayashi et al, 2011). KIF24 likewise suppresses cilia formation through the dual roles in which KIF24 recruits CP110 to the mother centriole and antagonizes the extension of the ciliary axoneme with its microtubule (MT)-depolymerizing activity in non-transformed cells (Kobayashi et al, 2011). NEK2 phosphorylates KIF24, which enhances MT-depolymerizing activity (Kim et al, 2015). MPP9 also forms a complex with CP110-CEP97-KIF24 to suppress ciliogenesis (Huang et al, 2018).

In this study, we generated KIF24-depleted PDAC cells to assess whether the forced expression of primary cilia restrained the proliferation of PDAC cells. Unexpectedly, we found that KIF24-depleted cells exhibited vigorous proliferation compared with control cells. KIF24 depletion was found to suppress the formation of multipolar spindles by clustering excess centrosomes and to improve mitotic progression in PDAC cells. On the other hand, the elimination of primary cilia in KIF24-depleted cells failed to affect both the proliferation and centrosome clustering. Mechanistically, NEK2-mediated phosphorylation and MT-depolymerizing activity were dispensable for the mitotic function of KIF24. The inhibition of HSET/KIFC1, which promotes the formation of centrosome clusters (Kleylein-Sohn et al, 2012), induced the de-clustering of centrosomes in KIF24-depleted cells. Moreover, KIF24 depletion specifically blocked the formation of multipolar spindles and induced hyper-proliferation in PDAC cells harboring supernumerary centrosomes. These results represent a novel function of KIF24 in the centrosome clustering, irrespective of primary ciliation in centrosome-amplified PDAC cells.

## Results

### KIF24 depletion induces primary cilia in PDAC cells

**Figure S1.**
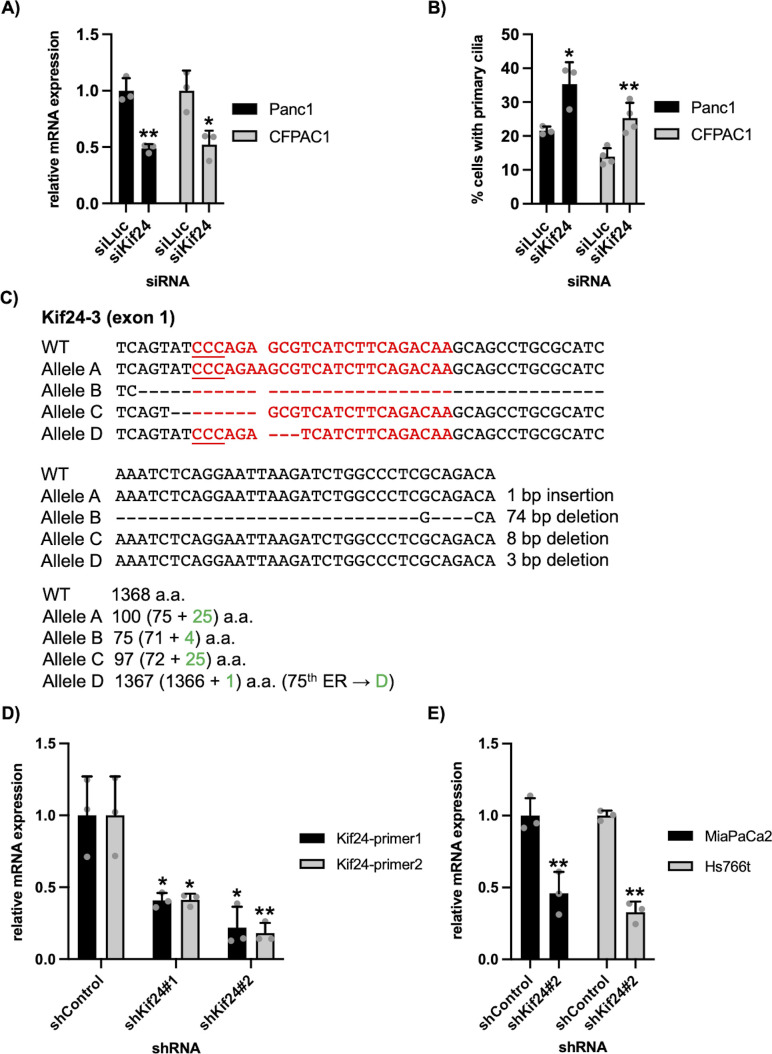
Mutation and expression of KIF24 in KIF24-depleted PDAC cells. **(A, B)** Panc1 or CFPAC1 cells transiently transfected with siLuciferase or siKif24 were cultured in serum-starved medium for 48 h. **(A)** Relative amount of KIF24 mRNA was determined using quantitative PCR. GAPDH was used as a control. Average of three independent experiments is shown. **(B)** Cells were immunostained as described in Fig 1B. The percentage of ciliated cells was determined. The average of three to four independent experiments is shown; >250 cells were scored each time. **(A, B, C, D)** (upper) Four different mutations (alleles A, B, C, and D) of the KIF24 gene in Kif24-3 Panc1 cells (note that Panc1 cells are frequently multiploid). Red sequences represent the target of gRNAs with underlined protospacer adjacent motif (PAM). (Lower) Proteins translated from each clone. Green shows incorrect residues. **(D, E)** shKif24-expressed Panc1 cells (D) or MiaPaCa2 and Hs766t cells (E) were cultured in serum-fed medium for 48 h. Relative amount of KIF24 mRNA was determined using quantitative PCR. GAPDH was used as a control. Average of three independent experiments is shown. **(B, E)** Kif24-primer2 pairs were used. **(A, B, D, E)** All data are shown as mean ± SD. Two-tailed *t* test. **P < 0.01; *P < 0.05.

**Figure 1.**
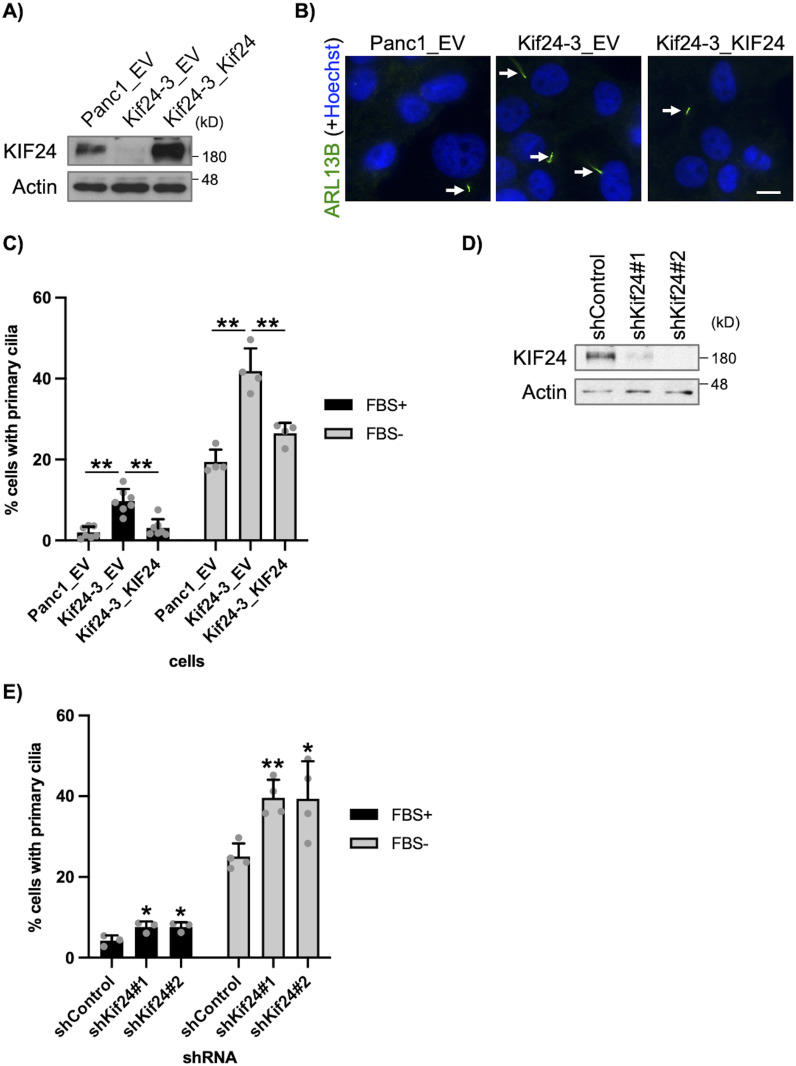
KIF24 depletion restores primary cilia in Panc1 cells. **(A)** The indicated Panc1 cells were cultured in serum-fed medium for 48 h. Cell extracts were immunoblotted with an anti-KIF24-2 antibody. β-Actin was used as a loading control. **(B, C)** The indicated Panc1 cells were cultured in serum-fed (FBS+) or serum-starved medium (FBS−) for 48 h and immunostained with an anti-Arl13b antibody (green). **(B)** Representative images of cells in FBS− cultivation. Arrows indicate primary cilia. DNA was stained with Hoechst (blue). Scale bar, 10 μm. **(C)** The percentage of ciliated cells was determined. The average of four to seven independent experiments is shown; >250 cells were scored each time. **(D)** The indicated Panc1 cells were cultured and immunoblotted as described in Fig 1A. **(E)** The indicated Panc1 cells were cultured and immunostained as described in Fig 1B. The percentage of ciliated cells was determined. The average of three to four independent experiments is shown; >250 cells were scored each time. **(C, E)** All data are shown as mean ± SD. Two-tailed *t* test. **P < 0.01; *P < 0.05. Source data are available for this figure.

To examine whether KIF24 contributes to the suppression of primary ciliogenesis in PDAC cells, we used Panc1 cells, which were originally isolated from a PDAC patient and assemble primary cilia with low frequency (Lieber et al, 1975; Nielsen et al, 2008). Because siRNA-mediated acute knockdown of KIF24 induced the formation of primary cilia in Panc1 cells (Fig S1A and B), KIF24-mutated Panc1 cells were subsequently generated using the CRISPR/Cas9 method. The sequencing analysis indicated four distinct mutations in Kif24-mutated cells (named Kif24-3 cells), leading to premature stop codons in three alleles (allele A–C) but an amino acid deletion in one allele (allele D) (Fig S1C), suggesting that KIF24 was not completely knocked out in Kif24-3 Panc1 cells. A rescue clone was further established in which ectopic KIF24 was stably expressed in Kif24-3 cells (Kif24-3_KIF24), and control clones harboring the empty vector (EV) (Panc1_EV and Kif24-3_EV) were also generated. Western blotting analysis showed KIF24 expression in Panc1_EV and Kif24-3_KIF24 cells but a substantial decrease in KIF24 in Kif24-3_EV cells (Fig 1A), indicating that KIF24 is drastically depleted in Kif24-3 cells. The assembly of primary cilia was assessed in these cells by immunofluorescence experiments using an anti-ARL13B antibody that specifically recognizes the ciliary membrane. Kif24-3 cells formed significantly more primary cilia than Panc1_EV and Kif24-3_KIF24 cells in both serum-fed (FBS+) and -deprived (FBS−) media (Fig 1B and C), suggesting that KIF24 suppresses primary ciliogenesis in Panc1 cells. In addition, Panc1 cells stably expressing shRNA targeting KIF24 were generated (Figs 1D and S1D). The percentage of cells with primary cilia was significantly increased in two individual shKIF24-expressing clones compared with shControl cells (Fig 1E). Furthermore, siRNA-mediated silencing of KIF24 in another PDAC cell line, CFPAC1, also induced ciliation (Fig S1A and B). These results collectively indicate that KIF24 negatively controls the assembly of primary cilia in PDAC cells, as expected from previous reports (Kobayashi et al, 2011; Kim et al, 2015).

### Loss of KIF24 enhances the proliferation of Panc1 cells in vitro and in vivo

**Figure 2.**
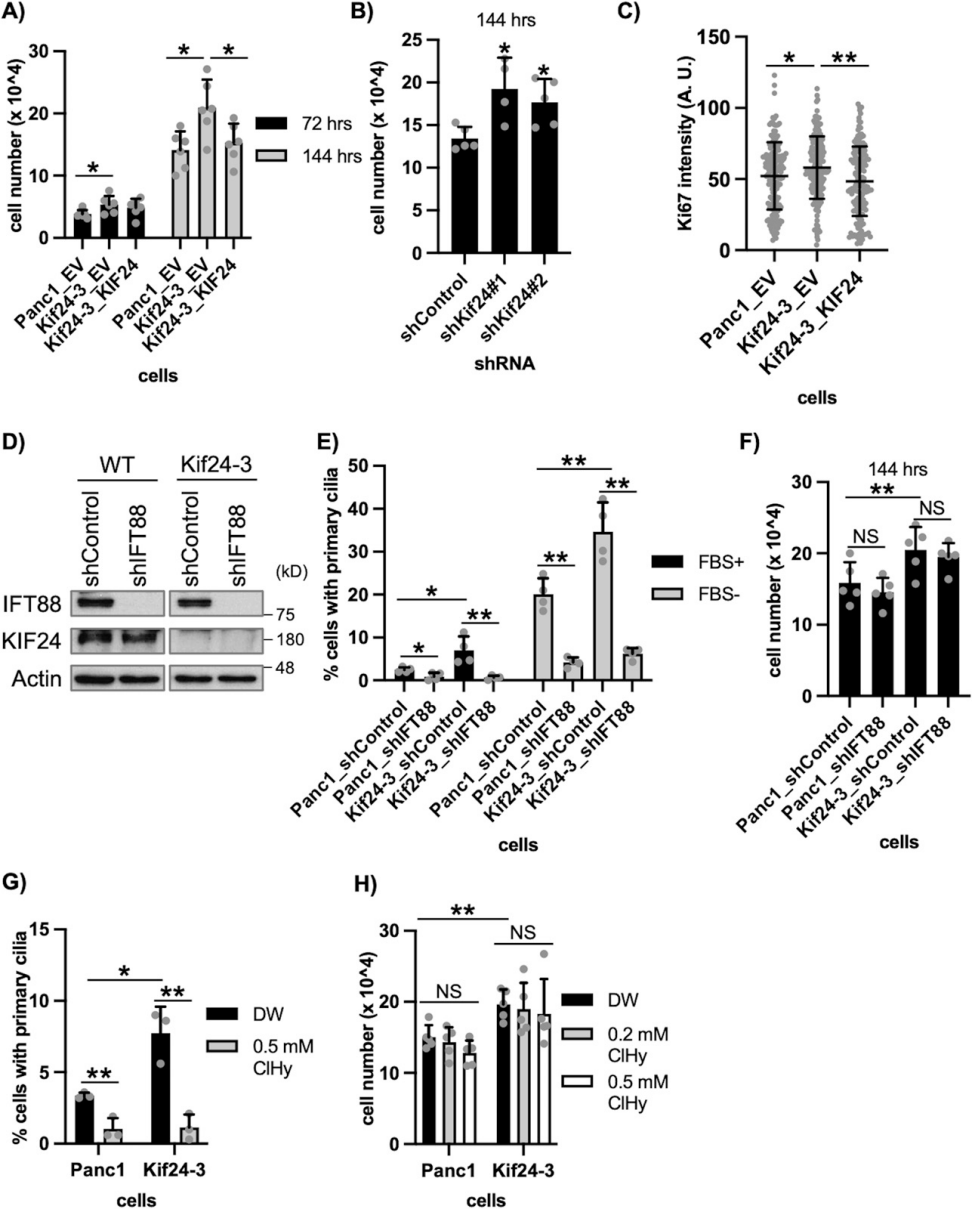
KIF24 depletion enhances proliferation of Panc1 cells in vitro. **(A, B)** The indicated Panc1 cells were cultured for 72 or 144 h, and the number of surviving cells was counted with a hemocytometer. The average of six (A) or four to five (B) independent experiments is shown. **(C)** The indicated Panc1 cells were cultured in serum-fed medium for 48 h and immunostained with an anti-Ki67 antibody. The quantified fluorescence intensity of Ki67 in the nucleus is shown. n = 160 (Panc1_EV), 160 (Kif24-3_EV), 153 (Kif24-3_KIF24). **(D)** The indicated Panc1 cells were cultured in serum-fed medium for 48 h, and their extracts were immunoblotted with anti-IFT88 and anti-KIF24-2 antibodies. β-Actin was used as a loading control. **(E)** The indicated Panc1 cells were cultured and immunostained as described in Fig 1B. The percentage of ciliated cells was determined. The average of four independent experiments is shown; >250 cells were scored each time. **(F)** The indicated Panc1 cells were cultured for 144 h, and the number of surviving cells was counted. The average of five independent experiments is shown. **(G)** The indicated Panc1 cells treated with distilled water (DW) or 0.5 mM ClHy were cultured in serum-fed medium for 48 h and immunostained as described in Fig 1B. The percentage of ciliated cells was determined. The average of three independent experiments is shown; >250 cells were scored each time. **(H)** The indicated Panc1 cells treated with DW or 0.5 mM ClHy were cultured for 144 h, and the number of surviving cells was counted. The average of five independent experiments is shown. **(A, B, C, E, F, G, H)** All data are shown as mean ± SD. Two-tailed *t* test. **P < 0.01; *P < 0.05. Source data are available for this figure.

The proliferation of KIF24-depleted cells was evaluated in vitro. As primary cilia appear to inhibit cell division, KIF24-depleted cells were predicted to exhibit slower growth because of an increase in primary cilia. However, to our surprise, both KIF24-mutated and KIF24-knockdown (KD) cells grew more vigorously than control cells (Fig 2A and B). Moreover, nuclear expression of Ki67, a marker of proliferating cells, was up-regulated in the KIF24-depleted cells (Fig 2C). These results suggest that KIF24 depletion promotes the proliferation of Panc1 cells.

**Figure S2.**
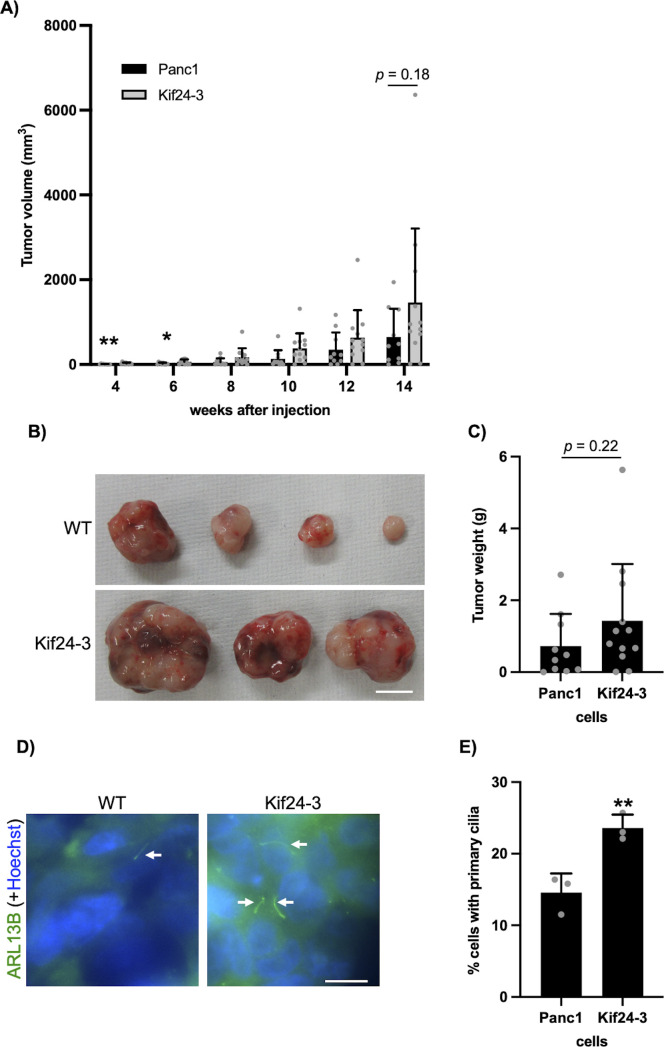
KIF24 mutation moderately promotes tumor formation in vivo. **(A, B, C)** The indicated Panc1 cells were injected into nude mice. n = 10 (WT), 12 (Kif24-3). **(A)** The volume of tumor was measured every 2 wk. **(B)** Excised tumors were imaged. Scale bar, 10 mm. **(C)** The weight of excised tumors was measured. **(D, E)** Tumor slices were immunostained as described in Fig 1B. **(D)** Arrows indicate primary cilia. DNA was stained with Hoechst (blue). Scale bar, 10 μm. **(E)** The percentage of ciliated cells was determined. The average of three independent experiments is shown; >250 cells were scored each time. **(A, C, E)** All data are shown as mean ± SD. Two-tailed *t* test. **P < 0.01; *P < 0.05.

Next, tumor formation in KIF24-mutated Panc1 cells was evaluated in a mouse xenograft model. Kif24-3-tumors were larger than tumors derived from parental cells at early stages (4 and 6 wk after injection) (Fig S2A). However, the differences in the tumor volume were not significant at later stages (8–14 wk after injection). Similarly, the excised Kif24-3 tumors weighed moderately heavier than the WT tumors (14 wk after injection) (Fig S2B and C). These results suggest that KIF24 depletion tends to accelerate tumor formation of Panc1 cells in vivo. Subsequently, the expression of primary cilia was examined in tumor slices. Consistent with previous results of in vitro cultivation (Fig 1B and C), more primary cilia were observed in the slices of the Kif24-3 tumors than in the WT tumors (Fig S2D and E). Altogether, these results suggest that KIF24 depletion in Panc1 cells induces modest enhancement of tumorigenesis and primary ciliogenesis in vivo.

### Primary cilia are irrespective of over-proliferation in KIF24-mutated cells

To test whether primary cilia are related to the hyper-proliferation of KIF24-depleted cells, an intraflagellar transport (IFT) protein IFT88, which is essential for cilia formation, was stably knocked down (Fig 2D). If forced ciliation by KIF24 loss is linked to the over-proliferation of Panc1 cells, de-ciliation might affect the over-proliferation. In contrast to the substantial decrease in primary ciliation (Fig 2E), cell growth was not affected by silencing of IFT88 in WT and Kif24-3 cells (Fig 2F). To further confirm the primary cilia-independent hyper-proliferation of Kif24-3 cells, cells were treated with chloral hydrate (ClHy), which is known to exclude primary cilia from the cell surface (Ho et al, 2013; Kobayashi et al, 2020). ClHy treatment significantly reduced ciliation but failed to impact growth in WT and Kif24-3 cells (Fig 2G and H). These results strongly indicate that KIF24 depletion induces over-proliferation of Panc1 cells independent of primary cilium assembly.

**Figure S3.**
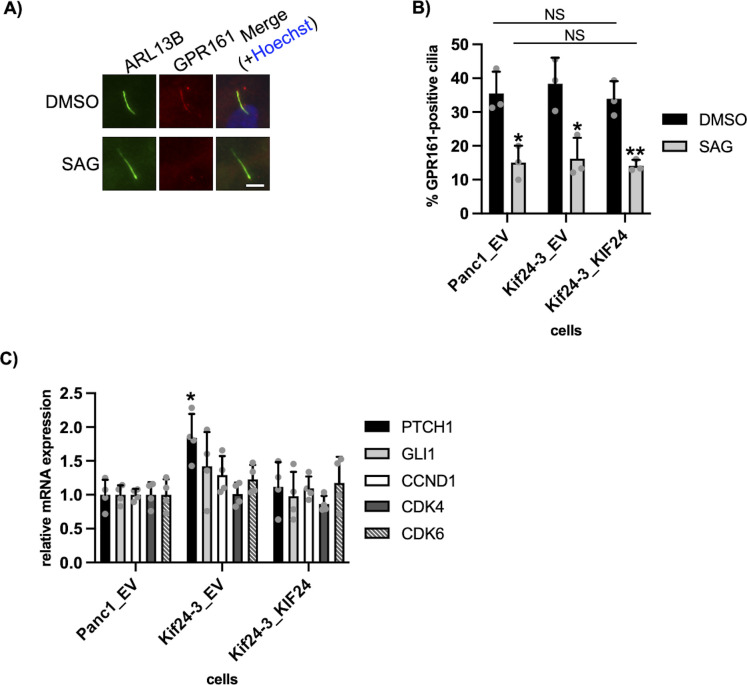
Hedgehog signaling in KIF24-depleted Panc1 cells. **(A, B)** The indicated Panc1 cells in serum-starved medium were treated with DMSO or 1 μM SAG for 48 h. Cells were immunostained with anti-ARL13B (green) and anti-GPR161 (red) antibodies. **(A)** DNA was stained with Hoechst (blue). Scale bar, 5 μm. **(B)** The percentage of cells with GPR161-positive cilia was determined. The average of three independent experiments is shown; >100 cells were scored each time. **(C)** The indicated cells were cultured in serum-fed medium for 48 h. Relative amounts of the indicated mRNA were determined using quantitative PCR. GAPDH was used as a control. Average of four independent experiments is shown. **(B, C)** All data are shown as mean ± SD. Two-tailed *t* test. NS, no significance.

As Hedgehog (Hh) signaling depends on primary cilia and is relevant in PDAC (Morris et al, 2010), we examined Hh signaling in KIF24-depleted cells. Ciliary localization of G-protein coupled receptor GPR161 and its reduction upon Smo agonist (SAG) stimulation were equally observed in Kif24-3 cells and WT or rescue cells (Fig S3A and B). These results suggest that primary cilia induced by KIF24 depletion are functional. Next, the expression of genes targeted by the Hh pathway was assessed using quantitative PCR (qPCR). Although the expression of patched 1/PTCH1 was elevated in Kif24-3 cells, the expression levels of the other genes were nearly similar in analyzed cells (Fig S3C), suggesting that the over-proliferation of KIF24-depleted cells was not because of enhanced Hh signaling.

### KIF24 depletion induces the centrosome clustering

**Figure S4.**
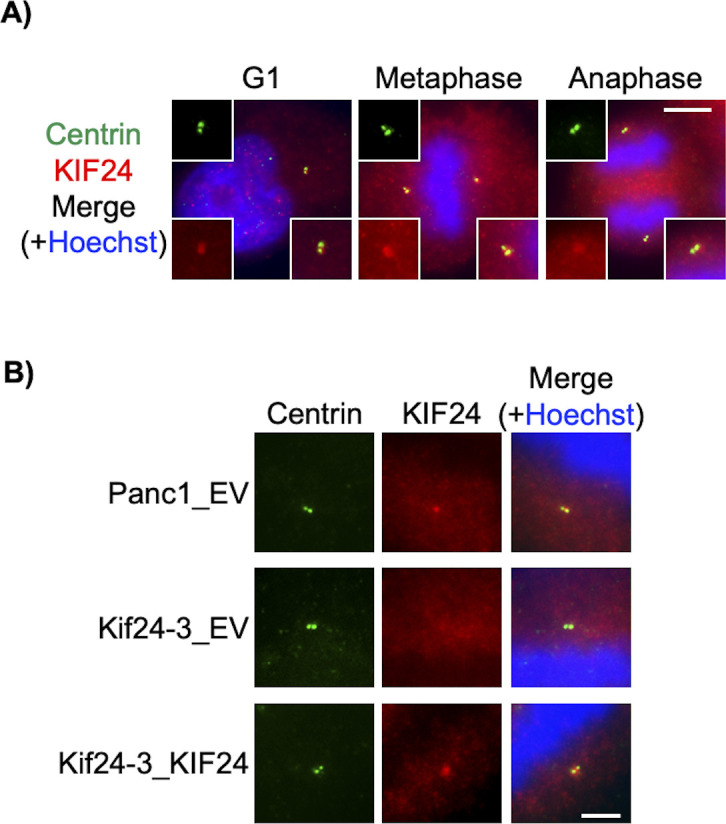
KIF24 localizes to centrosomes in Panc1 cells. **(A)** Panc1 cells were immunostained with anti-centrin (green) and anti-KIF24 (red) antibodies. DNA was stained with Hoechst (blue). Scale bar, 10 μm. **(B)** The indicated Panc1 cells were immunostained with anti-centrin (green) and anti-KIF24 (red) antibodies. DNA was stained with Hoechst (blue). Cells in the metaphase were imaged. Scale bar, 5 μm.

**Figure 3.**
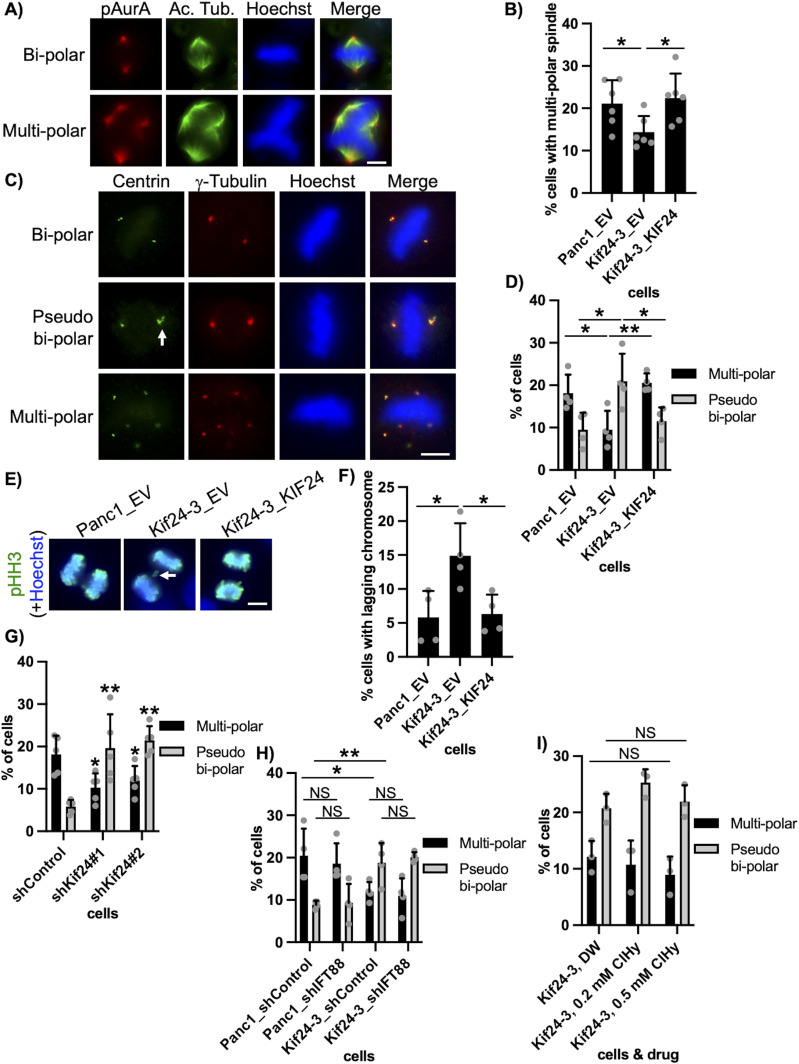
KIF24 depletion induces centrosome clustering in Panc1 cells. **(A, B)** The indicated Panc1 cells were immunostained with anti-acetylated tubulin (green) and anti-phosphorylated AurA (pAurA) (red) antibodies. **(A)** DNA was stained with Hoechst (blue). Scale bar, 10 μm. **(B)** The percentage of cells with multipolar spindles in the metaphase was determined. The average of six independent experiments is shown; >100 cells were scored each time. **(C, D)** The indicated Panc1 cells were immunostained with anti-centrin (green) and anti-γ-tubulin (red) antibodies. **(C)** DNA was stained with Hoechst (blue). Scale bar, 10 μm. **(D)** The percentage of cells with pseudo bipolar or multipolar spindles in the metaphase was determined. The average of four independent experiments is shown; >100 cells were scored each time. **(E, F)** The indicated Panc1 cells were immunostained with an anti-phosphorylated Histone H3 (pHH3) (green) antibody. **(E)** DNA was stained with Hoechst (blue). Scale bar, 10 μm. **(F)** The percentage of cells with lagging chromosome in the anaphase was determined. The average of four independent experiments is shown; >100 cells were scored each time. **(G, H)** The indicated Panc1 cells were immunostained and quantified as described in Fig 3C and D. The average of five (G) or four (H) independent experiments is shown; >100 cells were scored each time. **(I)** Kif24-3 cells treated with DW or ClHy were cultured in serum-fed medium for 48 h. Cells were immunostained and quantified as described in Fig 3C and D. The average of three independent experiments is shown; >100 cells were scored each time. **(B, D, F, G, H, I)** All data are shown as mean ± SD. Two-tailed *t* test. **P < 0.01; *P < 0.05; NS, no significance.

**Figure S5.**
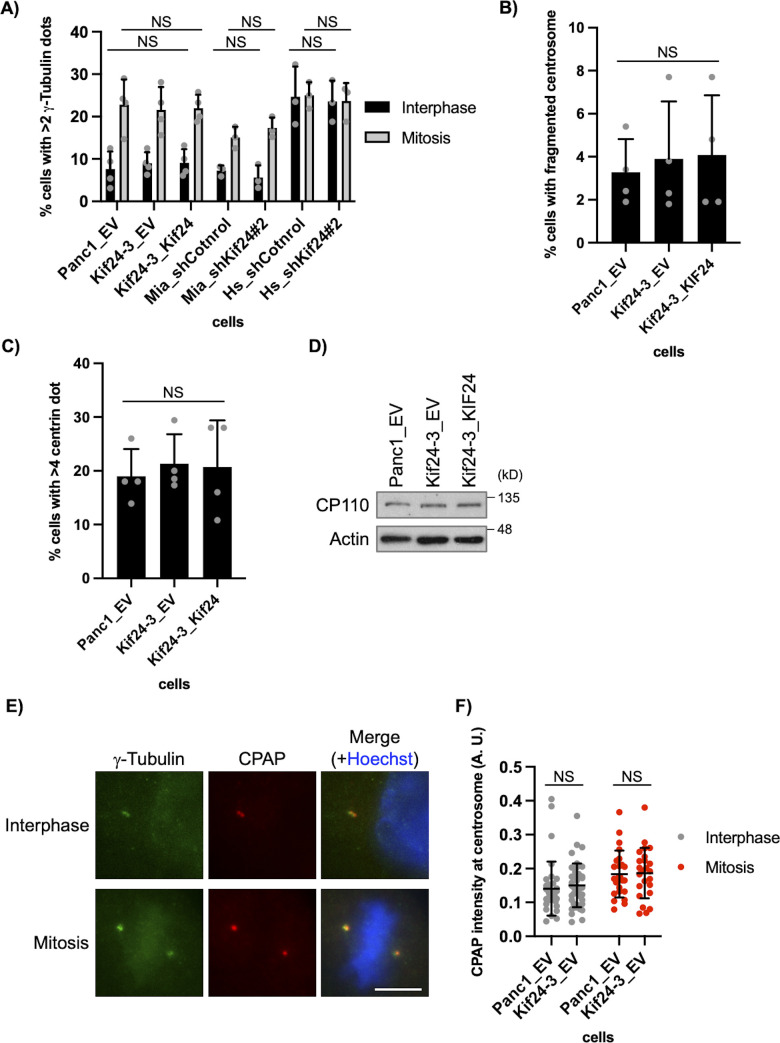
KIF24 depletion does not affect the centrosome number, centrosome fragmentation, and centriole duplication in Panc1 cells. **(A)** The indicated PDAC cells were immunostained with anti-γ-tubulin and anti-pHH3 antibodies. The percentage of cells with 2> γ-tubulin dots in the interphase (pHH3-negative) and mitosis (pHH3-positive) was determined. The average of three to four independent experiments is shown; >100 cells were scored each time. **(B, C)** The indicated Panc1 cells were immunostained as described in Fig 3C. **(B)** The percentage of cells with γ-tubulin-positive and centrin-negative dot in the metaphase was determined. The average of four independent experiments is shown; >100 cells were scored each time. **(C)** The percentage of cells with >4 centrioles in the metaphase was determined. The average of four independent experiments is shown; >100 cells were scored each time. **(D)** The indicated Panc1 cells were cultured in serum-fed medium for 48 h. Cell extracts were immunoblotted with an anti-CP110 antibody. β-Actin was used as a loading control. **(E, F)** The indicated Panc1 cells were immunostained with anti-γ-tubulin (green) and anti-CENPJ/CPAP (red) antibodies. **(E)** DNA was stained with Hoechst (blue). Scale bar, 10 μm. **(F)** The quantified fluorescence intensity of CPAP at centrosome is shown. Interphase, n = 34 (Panc1_EV), 41 (Kif24-3_EV); mitosis, n = 24 (Panc1_EV), 24 (Kif24-3_EV). **(A, B, C, D, F)** All data are shown as mean ± SD. Two-tailed *t* test. NS, no significance. Source data are available for this figure.

KIF24 is known to localize to the centrosome not only during the interphase but also during mitosis in mammalian cells (Kobayashi et al, 2011). We specifically detected KIF24 at the centrosome in Panc1 cells during mitosis (Fig S4A and B) and therefore focused on mitotic events in Kif24-3 cells. Immunofluorescence experiments to visualize mitotic morphologies showed that ∼20% of WT (Panc1_EV) cells form multipolar spindles (Fig 3A and B), which is consistent with a previous study showing that Panc1 cells frequently harbor amplified centrosomes and assemble multipolar spindles (Difilippantonio et al, 2009). In contrast, the proportion of mitotic cells with multipolar spindles was significantly decreased in Kif24-3_EV cells compared with Panc1_EV and Kif24-3_KIF24 cells (Fig 3A and B). Next, we stained cells with antibodies recognizing centrin and γ−tubulin to detect centrioles and centrosomes, respectively. Remarkably, this experiment revealed a decrease in multipolar cells with a concurrent increase in pseudo bipolar cells in which more than two centrin dots were observed on one side of the spindle (=γ-tubulin dot[s]) in mitotic Kif24-3 cells (Fig 3C and D), indicating that the supernumerary centrosomes were tightly clustered in KIF24-mutated cells. We also observed more mis-segregated chromosomes in the anaphase in Kif24-3 cells (Fig 3E and F), which is typically induced by pseudo bipolar spindles, supporting that the occurrence of mitotic cells with pseudo bipolar spindles was increased by KIF24 loss. Moreover, shKif24-Panc1 cells displayed more pseudo bipolar, rather than multipolar, spindles (Fig 3G). In contrast, the number of cells with increased γ-tubulin dots (>2 γ-tubulin dots) was not altered in Kif24-3 cells (Fig S5A). In addition, the percentages of mitotic cells with overamplified centrioles (>4 centrin dots) or fragmented centrosomes (γ-tubulin foci without centrin) were comparable among the analyzed cells (Fig S5B and C). Collectively, these results suggest that KIF24 depletion specifically evokes centrosome clustering.

Subsequently, immunofluorescence studies were conducted in cells treated with shIFT88 or ClHy. Neither IFT88-KD nor ClHy treatment affected the number of cells with multipolar and pseudo bipolar spindles in Kif24-3 cells (Fig 3H and I). These data suggest that the centrosome clustering occurs irrespective of primary cilia in KIF24-mutated cells, which is consistent with the over-proliferation of these cells.

### NEK2-mediated phosphorylation and MT-depolymerizing activity are dispensable for the mitotic function of KIF24

**Figure 4.**
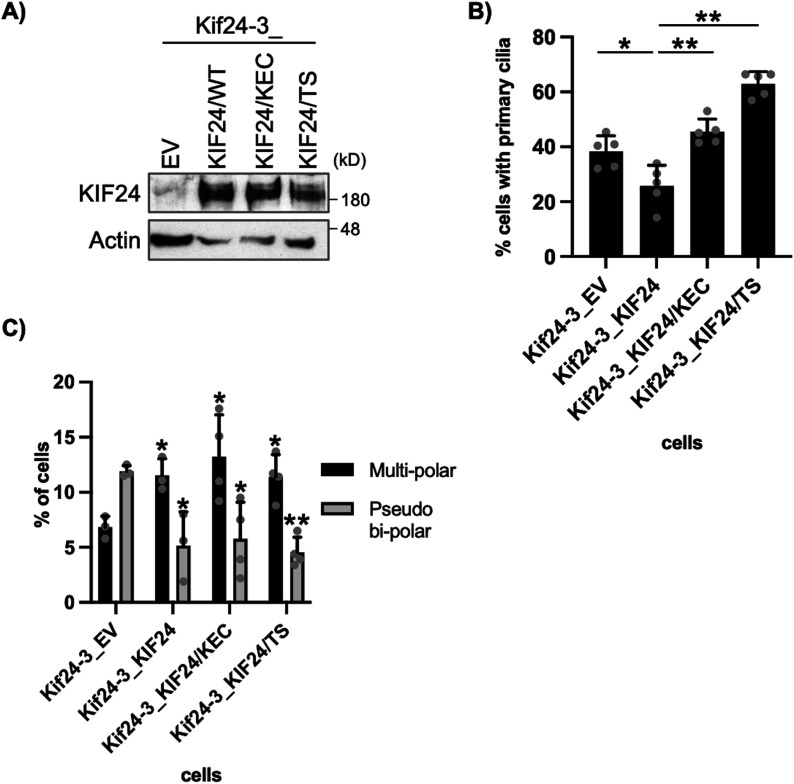
NEK2 phosphorylation or microtubule (MT)-depolymerizing activity dead mutants are sufficient for mitotic function of KIF24. **(A)** The indicated Panc1 cells were cultured and immunoblotted as described in Fig 1A. **(B)** The indicated Panc1 cells were cultured and immunostained as described in Fig 1B. The percentage of ciliated cells was determined. The average of five independent experiments is shown; >250 cells were scored each time. **(C)** The indicated Panc1 cells were cultured, immunostained, and quantified as described in Fig 3C and D. The average of three to four independent experiments is shown; >100 cells were scored each time. **(B, C)** All data are shown as mean ± SD. Two-tailed *t* test. **P < 0.01; *P < 0.05. Source data are available for this figure.

The amino acid residues KEC (positions 483–485) are conserved in the Kinesin-13 family of proteins and are important for the MT-depolymerizing activity of KIF24 (Kobayashi et al, 2011). In addition, the NEK2-mediated phosphorylation of Thr^622^ and Ser^623^ was found to enhance the MT-depolymerizing activity of KIF24 (Kim et al, 2015). KIF24/KEC483-485AAA (KEC)– or KIF24/TS622, 623AA (TS)–expressing Kif24-3 cells were generated to elucidate whether the modification and activation of KIF24 are involved in spindle morphology (Fig 4A). Although KIF24/KEC and KIF24/TS failed to suppress the assembly of primary cilia (Fig 4B), these mutants significantly decreased pseudo bipolar formation and concomitantly increased multipolar formation in KIF24-depleted cells (Fig 4C). These results suggest that the MT depolymerizing activity and NEK2-mediated phosphorylation of KIF24 are dispensable for its mitotic function and further support our idea that centrosome clustering occurs independently of primary cilium assembly in KIF24 depleted cells.

### HSET/KIFC1 inhibition suppresses centrosome clustering caused by KIF24 depletion

**Figure 5.**
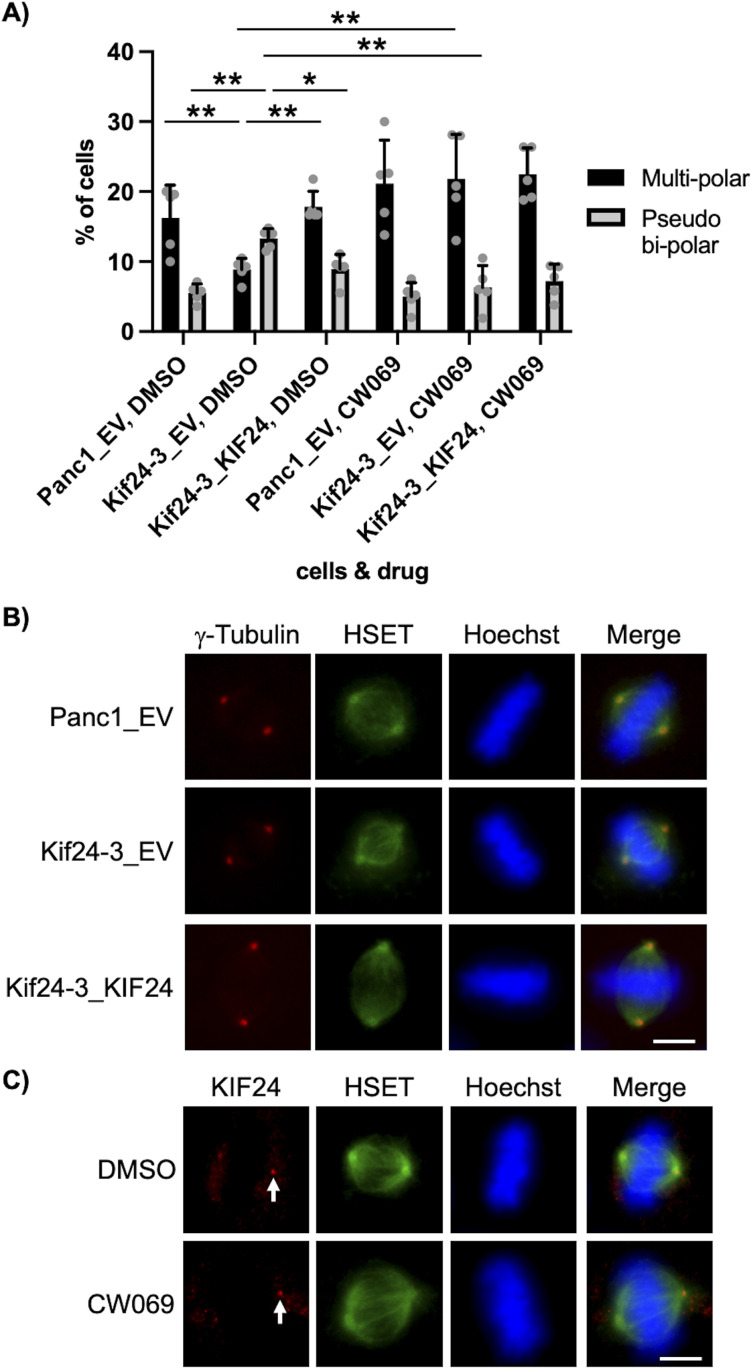
HSET/KIFC1 inhibition suppresses centrosome clustering induced by KIF24 depletion. **(A)** The indicated Panc1 cells treated with DMSO or 50 μM CW069 for 4 h were cultured, immunostained, and quantified as described in Fig 3C and D. The average of five independent experiments is shown; >100 cells were scored each time. **(B)** The indicated Panc1 cells were immunostained with anti-HSET/KIFC1 (green) and anti-γ-tubulin (red) antibodies. DNA was stained with Hoechst (blue). Scale bar, 10 μm. **(C)** Panc1 cells treated with DMSO or 50 μM CW069 for 4 h were immunostained with anti-HSET/KIFC1 (green) and anti-KIF24 (red) antibodies. Arrows indicate KIF24 at spindles. DNA was stained with Hoechst (blue). Scale bar, 10 μm. **(A)** All data are shown as mean ± SD. Two-tailed *t* test. **P < 0.01; *P < 0.05.

The mitotic kinesin HSET/KIFC1 promotes centrosome clustering (Kleylein-Sohn et al, 2012), and treatment with the allosteric HSET inhibitor CW069 provokes multipolar spindles in centrosome-amplified cells (Watts et al, 2013). These findings prompted us to examine whether HSET inhibition suppresses pseudo bipolar formation in KIF24-depleted cells. CW069 treatment clearly induced formation of multipolar spindles instead of pseudo bipolar assemblies in Kif24-3 cells, and the ratios were comparable in Panc1_EV and Kif24-3_KIF24 cells (Fig 5A). These results suggest that KIF24 functions upstream of HSET in the centrosome clustering cascade. On the other hand, HSET on the mitotic spindle microtubules was unaffected in KIF24-depleted cells (Fig 5B). KIF24 at spindles also remained unchanged by CW069 treatment (Fig 5C). These results suggest that KIF24 is unrelated to HSET localization and vice versa in mitosis.

CP110 expression was also evaluated because the silencing of KIF24 decreases CP110 protein levels in normal diploid RPE1 cells but not in cancerous U2OS cells (Kobayashi et al, 2011). We found that CP110 levels were not altered in Kif24-3 cells (Fig S5D), suggesting that, like in U2OS cells, CP110-dependent centriolar events are not affected by KIF24 depletion in Panc1 cancer cells. The centrosomal protein CENPJ/CPAP is involved in centrosome clustering in cancer cells (Mariappan et al, 2019) and antagonizes CP110 during the extension of centriole and cilia (Schmidt et al, 2009; Wu & Tang, 2012). Therefore, we examined CPAP foci at the centrosome in KIF24-depleted cells, but no alteration was detected (Fig S5E and F). These results suggest that KIF24 affects centrosome clustering independently of CP110 and CPAP.

### KIF24 depletion promotes mitotic progression in Panc1 cells

**Figure 6.**
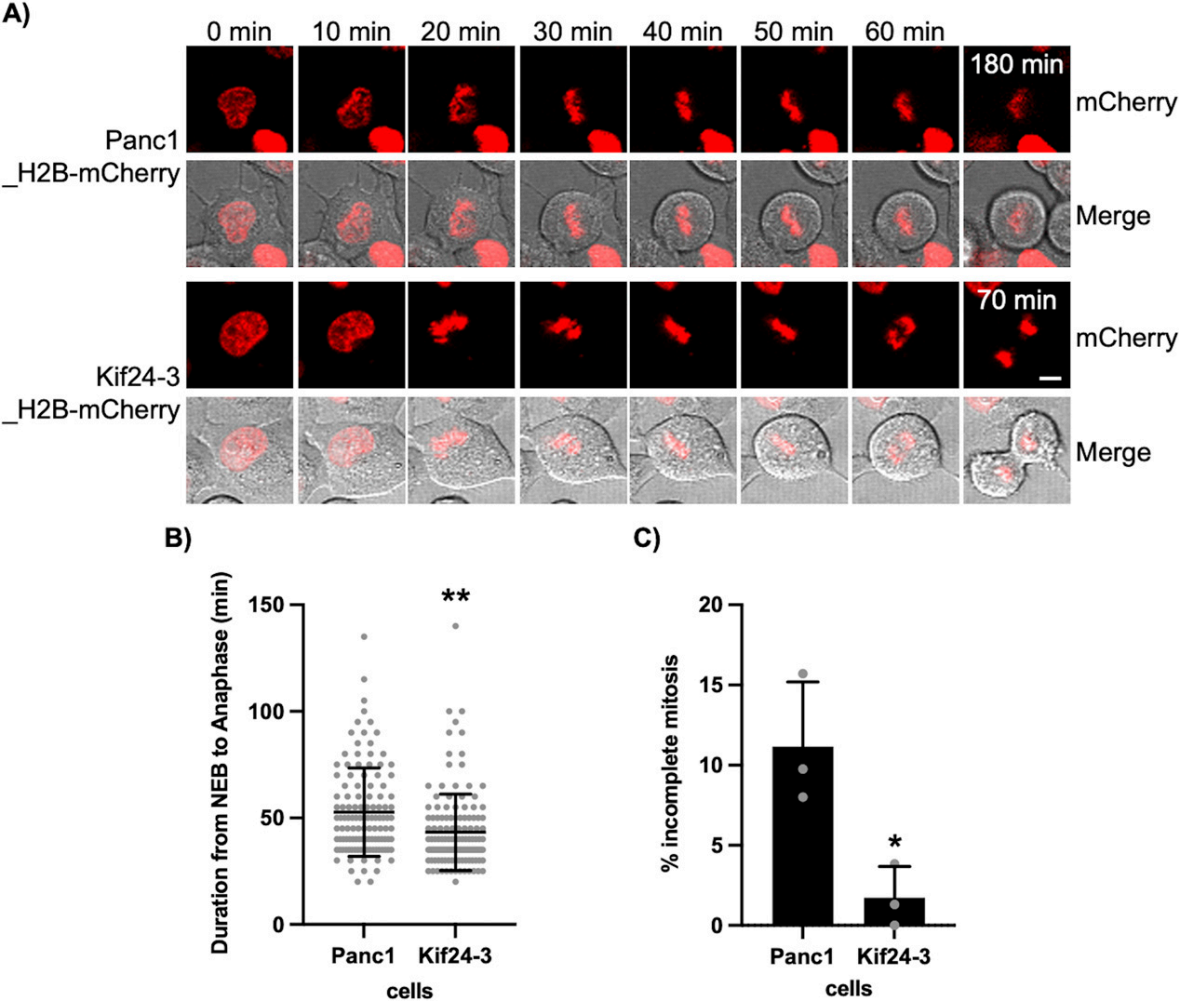
KIF24 depletion restores impaired mitotic events in Panc1 cells. **(A)** Frames from live cell imaging of Panc1 or Kif24-3 cells stably expressing H2B-mCherry. Scale bar, 10 μm. **(B)** Time from nuclear envelope breakdown to the anaphase onset in indicated cells was measured. n = 119 (Panc1_H2B-mCherry), 135 (Kif24-3_H2B-mCherry). **(C)** The percentage of cells that entered and remained in mitosis for >180 min. The average of three independent experiments is shown; >40 cells were scored each time. **(B, C)** All data are shown as mean ± SD. Two-tailed *t* test. **P < 0.01; *P < 0.05.

Live-cell imaging of mitotic progression was subsequently performed in KIF24-mutated cells. To visualize the chromosome dynamics during mitosis, histone H2B-mCherry–stably expressing Panc1 or Kif24-3 cells were generated. Time-lapse imaging revealed that the mitotic duration from nuclear envelope breakdown to the anaphase onset was slightly but significantly shortened in Kif24-3 cells (Fig 6A and B). Moreover, the ratio of cells that entered mitosis but failed to divide was largely decreased in Kif24-3 cells (Fig 6A and C). These results suggest that KIF24 depletion improves the mitotic progression in centrosome-amplified Panc1 cells, probably leading to their accelerated proliferation.

### KIF24 depletion suppresses multipolar spindle formation and enhances cell growth specifically in centrosome-amplified PDAC cells

**Figure 7.**
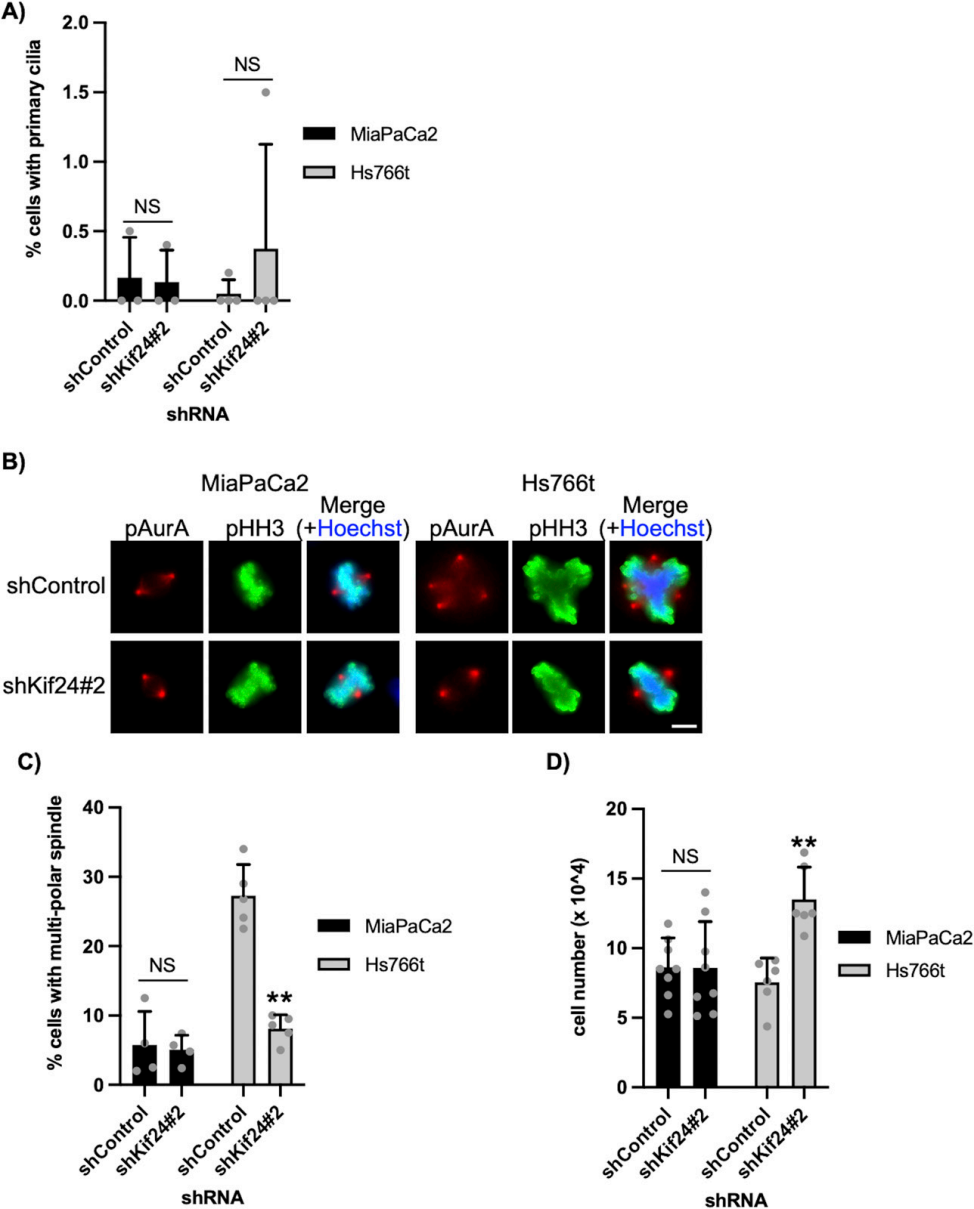
KIF24 depletion suppresses multipolar spindle formation and promotes cell growth in centrosome-amplified PDAC cells. **(A)** The indicated PDAC cells were cultured and immunostained as described in Fig 1B. The percentage of ciliated cells was determined. The average of three (MiaPaCa2) or four (Hs766t) independent experiments is shown; >250 cells were scored each time. **(B, C)** The indicated PDAC cells were immunostained with anti-pHH3 (green) and anti-pAurA (red) antibodies. **(B)** DNA was stained with Hoechst (blue). Scale bar, 10 μm. **(C)** The percentage of cells with multipolar spindles in the metaphase was determined. The average of four (MiaPaCa2) or five (Hs766t) independent experiments is shown; >100 cells were scored each time. **(D)** The indicated cells were cultured for 96 h (MiaPaCa2) or 192 h (Hs766t), and the number of surviving cells was counted with a hemocytometer. The average of eight (MiaPaCa2) or six (Hs766t) independent experiments is shown. **(A, C, D)** All data are shown as mean ± SD. Two-tailed *t* test. **P < 0.01; *P < 0.05; NS, no significance.

To test whether KIF24 controls centrosome clustering in other PDAC cells, shKIF24-expressing MiaPaCa2 or Hs766t cells were generated (Fig S1C). A previous report indicated that centrosomes are amplified in Hs766t cells but not in MiaPaCa2 cells (Difilippantonio et al, 2009). These PDAC cells rarely assembled primary cilia even after KIF24 silencing, suggesting that these cells have essentially lost the ability to ciliate (Fig 7A). In MiaPaCa2 cells, both control and KIF24-depleted cells assembled multipolar spindles with only ∼5% frequency (Fig 7B and C). In contrast, multipolar spindles were detected in ∼27% of control Hs766t cells, and this frequency was considerably reduced by KIF24 depletion (Fig 7B and C). Concomitant with multipolar spindle formation, KIF24-depleted Hs766t cells grew more vigorously than control Hs766t cells, in sharp contrast with MiaPaCa2 cells (Fig 7D). On the other hand, the number of cells with increased γ-tubulin dots was comparable between control and KIF24-depleted PDAC cells (Fig S5A). These results suggest that KIF24 induces multipolar spindle formation and thereby slow growth, specifically in centrosome-amplified PDAC cells.

## Discussion

This study analyzed PDAC cells with supernumerary centrosomes revealing that KIF24 suppresses the clustering of excess centrosomes during mitosis and the assembly of primary cilia. NEK2-mediated phosphorylation and MT-depolymerizing activity were dispensable for the mitotic role of KIF24, unlike the ciliary role, indicating that distinct regulatory systems govern the dual functions of KIF24. These mitotic and ciliary phenotypes in cells depleted of KIF24 are thought to exert reciprocal effects on cell proliferation. KIF24-depleted PDAC cells overgrew in vitro, in which primary cilia are rarely assembled (∼10% in Kif24-3 cells, Fig 1C), probably because of improved mitosis. The tumor development also occurred earlier with KIF24-depleted cells than with control cells in vivo (4–6 wk after injection), suggesting that the mitotic effect of KIF24 depletion promotes the tumor onset. In contrast, Kif24-3 tumors were not significantly larger than those derived from parental cells in the late stages (8–14 wk after injection). As more primary cilia were assembled in the excised tumors than in the cultured cells (∼25% in Kif24-3 tumor slices, Fig S2E), it is plausible that these organelles opposed the long-term in vivo growth of Kif24-3 cells. Alternatively, aneuploidy provoked by chromosome mis-segregation in Kif24-3 cells may result in heterogeneous populations of cells with diverse proliferative profiles during long-term tumor growth. This notion is supported by data showing that Kif24-3 tumors exhibited larger variation than wild-type tumors. On the other hand, as the transient assembly of primary cilia can promote cell proliferation by activating the Hh pathway in medulloblastoma cells (Ho et al, 2020), we cannot exclude the possibility that primary cilia induced by KIF24 loss positively affects tumor growth in vivo, where implanted tissue is presumably exposed to Hh ligands. In that sense, an earlier onset of Kif24-3 tumors may be attributed to increased number of primary cilia. A clinical report showing that expression of primary cilia is correlated with poor prognosis of PDAC patients also suggests that primary cilia could promote growth in PDAC in vivo (Emoto et al, 2014). Given that primary cilia appear to be dynamically assembled and disassembled even in KIF24-depleted cells, perturbation of their disassembly may lead to different outcomes in PDAC growth.

Several MT-binding proteins are known to be involved in the regulation of multipolar spindle formation in cancer cells but not in PDAC cells. HSET promotes the clustering of amplified centrosomes in breast cancer and melanoma cells (Kleylein-Sohn et al, 2012). HSET interacts with a centrosomal protein CEP215 during its operation (Chavali et al, 2016). IFT-B proteins, which are essential for cilia assembly, are also associated with HSET to promote centrosome clustering in several centrosome-amplified cells (Vitre et al, 2020), whereas we did not detect any alterations of centrosome clustering in IFT88-depleted Panc1 cells. Contrary to CEP215 and IFT-B proteins, KIF24 is unlikely to directly associate with HSET but probably acts upstream of HSET in PDAC cells based on our data. Another kinesin, KIF18A, facilitates the proliferation of colorectal and breast cancer cells through the regulation of centrosome fragment formation (Marquis et al, 2021). The association of CPAP with MT is required for centrosome clustering in breast and lung cancer cells (Mariappan et al, 2019). CPAP antagonizes CP110 in centriole elongation and is required for ciliogenesis (Schmidt et al, 2009; Wu & Tang, 2012). KIF24 cooperatively interacts with CP110 to suppress primary cilia formation (Kobayashi et al, 2011). Although the expression of CP110 and CPAP was not affected by KIF24 depletion, future studies are needed to examine whether KIF24-CP110 is involved in the functionality of CPAP.

Although we showed that primary cilium assembly has no bearing on centrosome clustering in PDAC cells, the possibility that primary cilia or their remnants after resorption influence mitosis cannot be excluded. Pharmacological inhibition of mitotic kinases, Aurora A or PLK1, induces mitotic primary cilia in normal mouse IMCD3 cells (Bowler et al, 2019). It was recently reported that NEK2 knockout causes the expression of primary cilia or their remnants in mitotic RPE1 cells (Viol et al, 2020). As described above, NEK2 phosphorylates and activates KIF24 to disassemble primary cilia in the G2–M phase (Kim et al, 2015). Indeed, mitotic primary cilia in KIF24-mutated Panc1 cells were found; however, we did not observe detectable alterations in centrosome clustering (data not shown). It will be interesting to determine whether and how primary cilia influence various mitotic events in cancer cells.

Centrosome clustering is an attractive target for cancer therapy because it is frequently observed in cancer cells but not in normal cells. Based on our study, potentiation of the mitotic activity of KIF24 could be a valid intervention strategy for PDAC, although we need to consider that loss of primary cilia by KIF24 might aggravate this cancer at present. Future studies clarifying the mechanistic details of KIF24 in spindle formation will represent a promising therapeutic target to develop novel centrosome de-clustering drugs for PDAC.

## Materials and Methods

### Cell culture

Panc1 (American Type Culture Collection), MiaPaCa2, Hs766t (Li et al, 2013), Kif24-3 Panc1 (this study), and Lenti-X 293T (gift from M Hagiwara) cells were grown in DMEM (Nacalai Tesque) supplemented with 10% FBS (Biosera) and 100 U/ml penicillin and 100 μg/ml streptomycin (P/S) (Nacalai Tesque). CFPAC1 (American Type Culture Collection) cells were grown in IMDM (Nacalai Tesque) supplemented with 10% FBS and P/S.

### Antibodies and reagents

Antibodies used in this study include mouse anti-glutamylated tubulin (GT335) (1:1,000 [IF], AG-20B-0020; Adipogen), rabbit anti-ARL13B (1:1,000 [IF], 17711-1-AP; Proteintech), mouse anti-ARL13B (1:1,000 [IF], 75-287; NeuroMab), rabbit anti-KIF24 (1:200 [IF] [Kobayashi et al, 2011]), rabbit anti-KIF24-2 (1:500 [WB], this work), rabbit anti-CP110 (1:1,000 [WB] [Chen et al, 2002]), mouse anti-phosphorylated AurA (1:100 [IF], #3079; Cell Signaling), mouse anti-acetylated tubulin (1:1,000 [IF], T7451; Sigma-Aldrich), goat anti-γ-tubulin (1:400 [IF], sc-7396; Santa Cruz), mouse anti-centrin (1:1,000 [IF], 04-1624; Millipore), mouse anti-phosphorylated Histone H3 (1:1,000 [IF], MABI0012; MAB Institute), rabbit anti-IFT88 (1:1,000 [WB], 13967-1-AP; Proteintech), mouse anti-KIFC1/HSET (1:500 [IF], sc-100947; Santa Cruz), rabbit anti-Ki67 (1:2,000 [IF], ab15580; Abcam), rabbit anti-CENPJ/CPAP (1:200 [IF], 11517-1-AP; Proteintech), rabbit anti-GPR161 (1:100 [IF], 13398-1-AP; Proteintech), and mouse anti-β-Actin (1:1,000, sc-47778; Santa Cruz). A rabbit anti-KIF24-2 antibody was produced by immunizing a GST fusion protein containing residues 1,201–1,368 of KIF24 into rabbits and purified as described previously (Kobayashi et al, 2011). Reagents used in this study include chloral hydrate (ClHy) (07922-62; Nacalai Tesque), thymidine (89270; Sigma-Aldrich), CW069 (S7336; Selleckchem), Smo agonist (SAG) (1939; BioVision), and Hoechst 33342 (04915-82; Nacalai Tesque).

### Plasmids

To generate gRNA targeting KIF24, an annealed oligo was inserted into pSpCas9(BB)-2A-Puro (PX459) V2.0 (Addgene) (Ran et al, 2013). To generate shRNA targeting IFT88, KIF24, or negative control, annealed oligo was inserted into pLKO.1 (Addgene) (Stewart et al, 2003). The oligo DNAs are listed in Table S1. To generate KIF24 wild-type or KEC483-485AAA (KEC), human KIF24 fragments encoding residues 1–4,383 were excised from pEGFP-C1-KIF24 (Kobayashi et al, 2011) and sub-cloned into pLVX-IRES-Puro (Clontech). The KIF24/TS621-622AA (TS) construct was prepared using PCR-based mutagenesis with the primers listed in Table S1. To generate H2B-mCherry, a human H2BC11 fragment encoding residues 1–381 was amplified using PCR with the primers listed in Table S1 and then sub-cloned into pLVX-mCherry-IRES-Puro. pLVX-mCherry-IRES-Puro was constructed by replacing GFP of pLVX-GFP-IRES-Puro with mCherry (Kim et al, 2015).

Table S1. DNA oligonucleotides used in this study.

Plasmid transfection into Panc1 and Lenti-X 293T cells was performed using Lipofectamine 2000 (Invitrogen) and PEI Max (Polysciences) according to the manufacturer’s instructions, respectively.

### Generation of Kif24-3 cells

The PX459-KIF24 plasmid was transfected into Panc1 cells using Lipofectamine 2000. Transfected cells were cultured in medium containing 5 μg/ml puromycin (Nacalai Tesque) for 72 h and singly plated into 96-well plates. Genomic DNA was extracted from surviving cells using QuickExtract DNA Solution 1.0 (Epicentre), and amplified PCR products using the primers listed in Table S1 were sub-cloned into pGEM-T Easy (Promega). The purified plasmid DNA was sequenced using M13 primers.

### Generation of stable cells

The lentivirus supernatant was produced by co-transfection of pLVX-IRES-Puro (EV), pLVX-KIF24-IRES-Puro (KIF24), pLVX-KIF24/TS-IRES-Puro (KIF24/TS), pLVX-KIF24/KEC-IRES-Puro (KIF24/KEC), pLVX-H2B-mCherry-IRES-Puro (H2B-mCherry), pLKO.1-shControl, pLKO.1-shKif24#1, pLKO.1-shKif24#2, or pLKO.1-shIFT88 with the Δ8.9, pcRev, and VSVG plasmids (gift from M Hagiwara) into Lenti-X 293T cells using PEI Max. The virus supernatant was harvested at 72 h post-transfection and concentrated using a Lenti-X Concentrator (Clontech). Panc1, MiaPaCa2, and Hs766t cells were incubated with virus in the presence of 5 μg/ml polybrene (Nacalai Tesque) for 72 h. The infected cells were subsequently cultured in medium containing 3 μg/ml (Panc1), 0.5 μg/ml (MiaPaCa2), or 1.5 μg/ml (Hs766t) puromycin for 8–20 d. Established cells were cultured in medium with puromycin. Panc1/WT_EV cells were generated previously (Kobayashi et al, 2020).

### RNA interference

The siRNA oligos used in this study were siLuciferase (5′-CGUACGCGGAAUACUUCGAuu-3′; Sigma-Aldrich) and siKIF24 (5′-GGAAGAAAGCUCCGAAAUAuu-3′; Sigma-Aldrich). siRNA (20 pmol) was transfected into Panc1 or CFPAC1 cells in a 24-well plate using Lipofectamine RNAiMAX (Invitrogen) according to the manufacturer’s instruction.

### Cell growth assay

1 × 10^4^ cells were seeded into 24-well plate and cultured for 72 or 144 h (Panc1), 96 h (MiaPaCa2), and 192 h (Hs766t). The number of trypsinized cells was counted using a hemocytometer.

### Western blotting

Cells were lysed with lysis buffer (50 mM Hepes-NaOH, pH 7.5, 150 mM NaCl, 5 mM EDTA, 0.5% NP-40, 10% glycerol, 1 mM DTT, 0.5 mM PMSF, 2 μg/ml leupeptin, 5 mM NaF, 10 mM β-glycerophosphate, and 1 mM Na_3_VO_4_) at 4°C for 30 min. A 20 μg lysate was loaded and analyzed using SDS–PAGE and immunoblotting.

### Immunofluorescence microscopy

Cultured cells were fixed with cold methanol for 5 min, 4% paraformaldehyde (Nacalai Tesque)/PBS for 10 min, or 3.7% formalin (Nacalai Tesque)/PBS for 10 min. After permeabilization with 0.2% Triton X-100/PBS for 10 min, the slides were blocked with 5% BSA/PBS before incubation with the primary antibodies. Primary and secondary antibodies were diluted to the desired concentrations using 5% BSA/PBS. As secondary antibodies, we used Alexa Fluor 488– or Alexa Fluor 594–conjugated donkey anti-mouse, anti-rabbit, or anti-goat IgG (Invitrogen). The cells were stained with Hoechst 33342 to visualize DNA. Slides mounted with PermaFluor Mounting Medium (Thermo Fisher Scientific) were observed and imaged using Axio Observer with a 63× lens.

Tumors were fixed with 3.7% formalin/PBS at 4°C for 12 h; sequentially equilibrated with 10%, 20%, and 30% sucrose/PBS; and embedded with OCT compound (Sakura Finetek) at −80°C. The frozen tumors were sliced into 10 μm-thick sections using Cryostat NX70 (Thermo Fisher Scientific) and mounted on MAS-coated slide (Matsunami). The mounted sections were fixed with Acetone for 15 min, soaked into boiled water for 15 min to retrieve antigens, and permeabilized with 0.2% Triton X-100/PBS for 10 min. After permeabilization, procedures were same as those used for the cultured cells. Fluorescence was quantified using ImageJ/Fiji software (Kobayashi et al, 2020).

### qPCR

Total RNA was isolated from cultured cells using Sepasol (Nacalai Tesque), and following reverse transcription reaction was performed using a ReverTra Ace qPCR RT kit (TOYOBO). qPCR was performed using THUNDERBIRD SYBR qPCR mix (TOYOBO) in a LightCycler96 (Roche). All reactions were conducted according to the manufacturer’s instructions. The primers are listed in Table S1.

### Live cell imaging

H2B-mCherry–expressing Panc1 or Kif24-3 cells were seeded into a CELLview Cell Culture Dish (Greiner) and treated with 2 mM thymidine for 40 h. At 9 h after thymidine washout, the cells were imaged using an LSM710 (Zeiss) operated by Zen software (Zeiss). Six Z-stack images were acquired every 5 min for 8 h using a 20× objective lens (Zeiss).

### Xenograft

2 × 10^6^ Panc1 cells in PBS were subcutaneously injected into 6-wk-old female nude mouse (Nihon SLC). Tumors were measured each week using a caliper, and their volumes were calculated using the formula: length × width × height × 0.5. After 14 wk, the tumors were excised and weighed. All experiments were approved by the NAIST Animal Committee and conducted in accordance with the guideline of the NAIST animal facility.

### Statistical analysis

The statistical significance of the differences was determined using two-tailed *t* test. The figure legends indicate the number of independent replicates conducted and the number of cells analyzed in each replicate. Differences were considered as significant when *P* < 0.05.

## Supplementary Material

Reviewer comments
