# Supplementary figures and images for: KIF24 depletion induces clustering of supernumerary centrosomes in PDAC cells
Source: Life Sci Alliance. 2022 Jul 8;5(11):e202201470. doi: 10.26508/lsa.202201470 (PMC9270500; doi:10.26508/lsa.202201470)

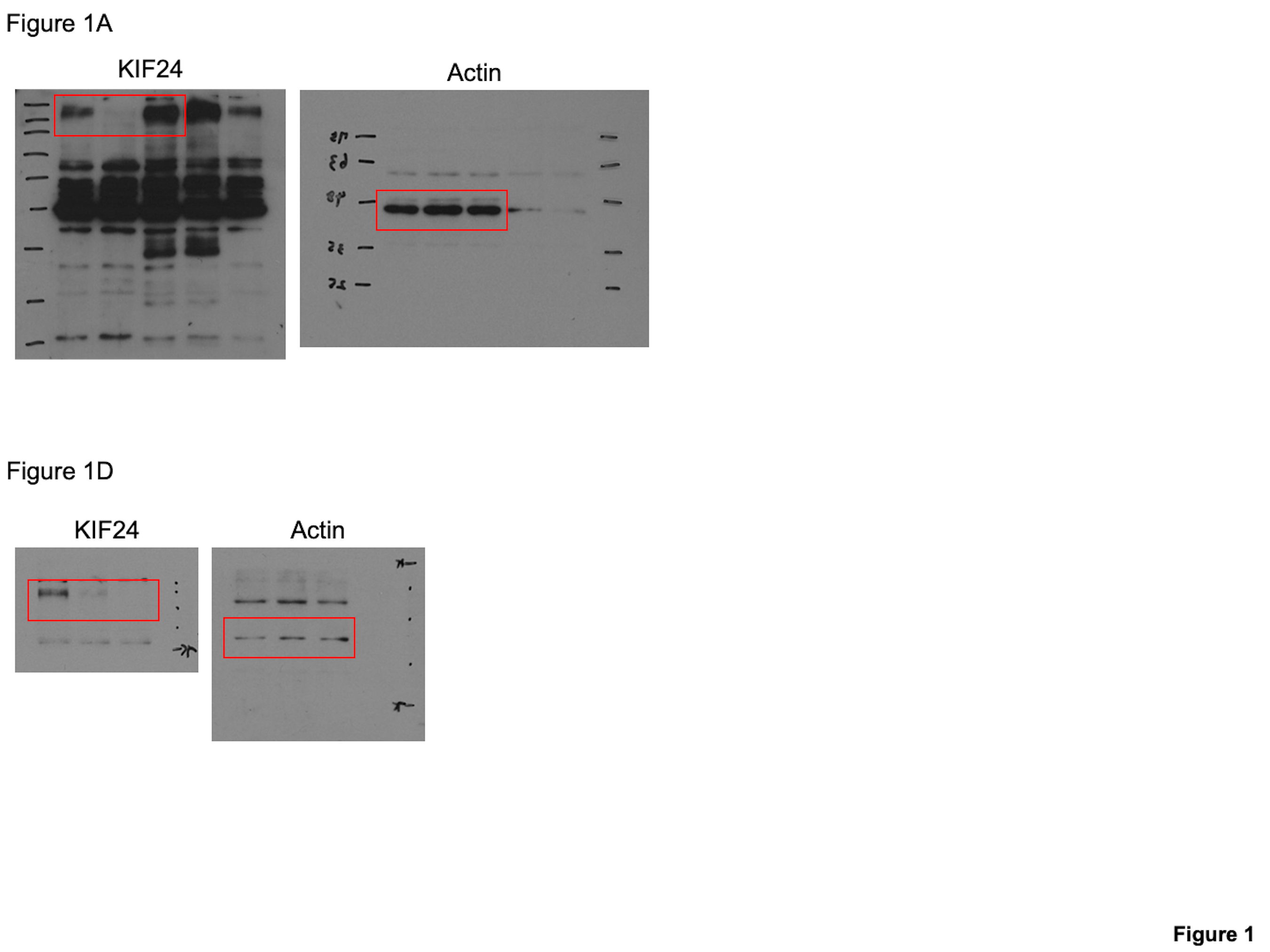

Supplement: Supplementary file 1 [file LSA-2022-01470_SdataF1.tif]

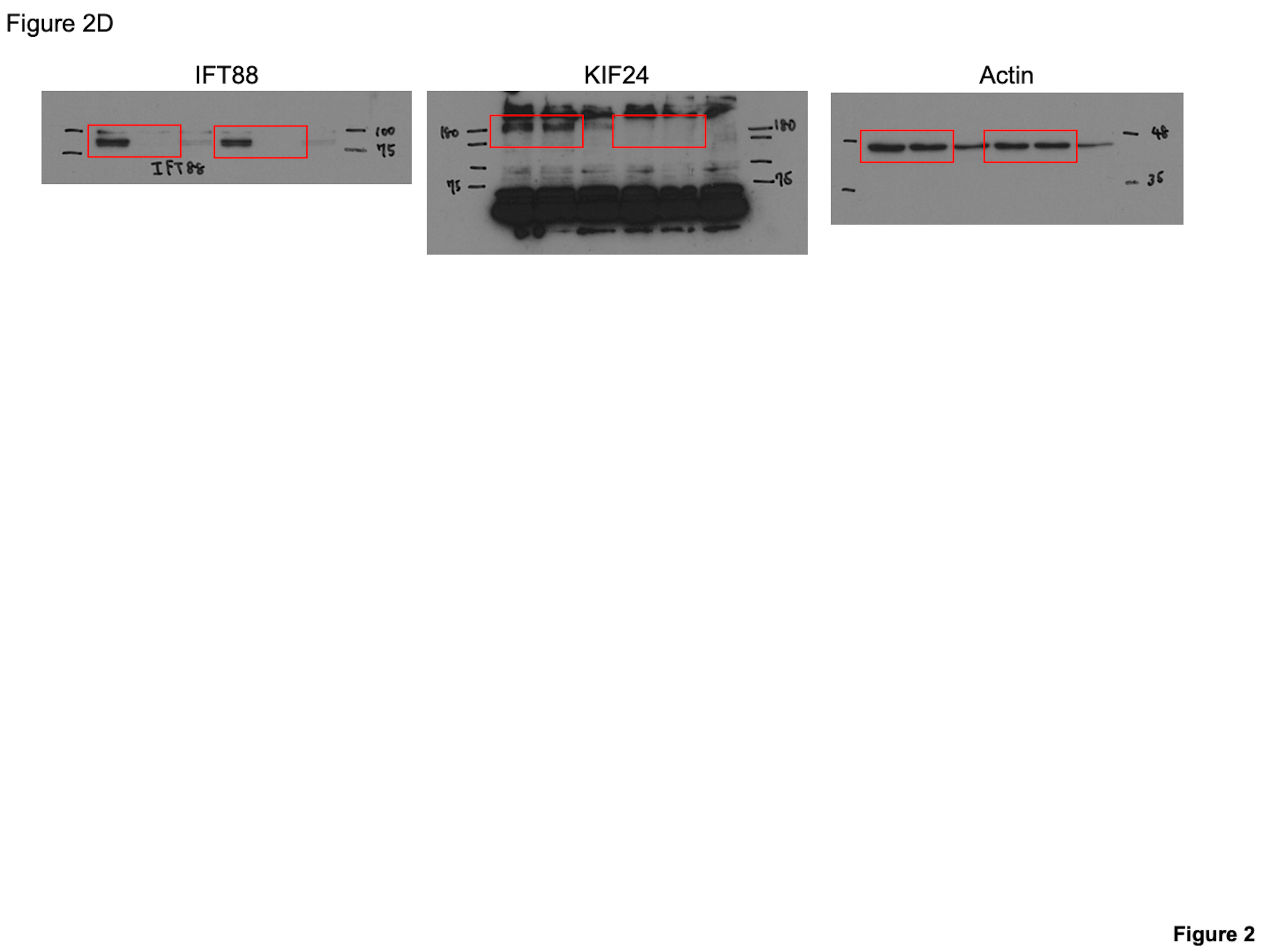

Supplement: Supplementary file 2 [file LSA-2022-01470_SdataF2.tif]

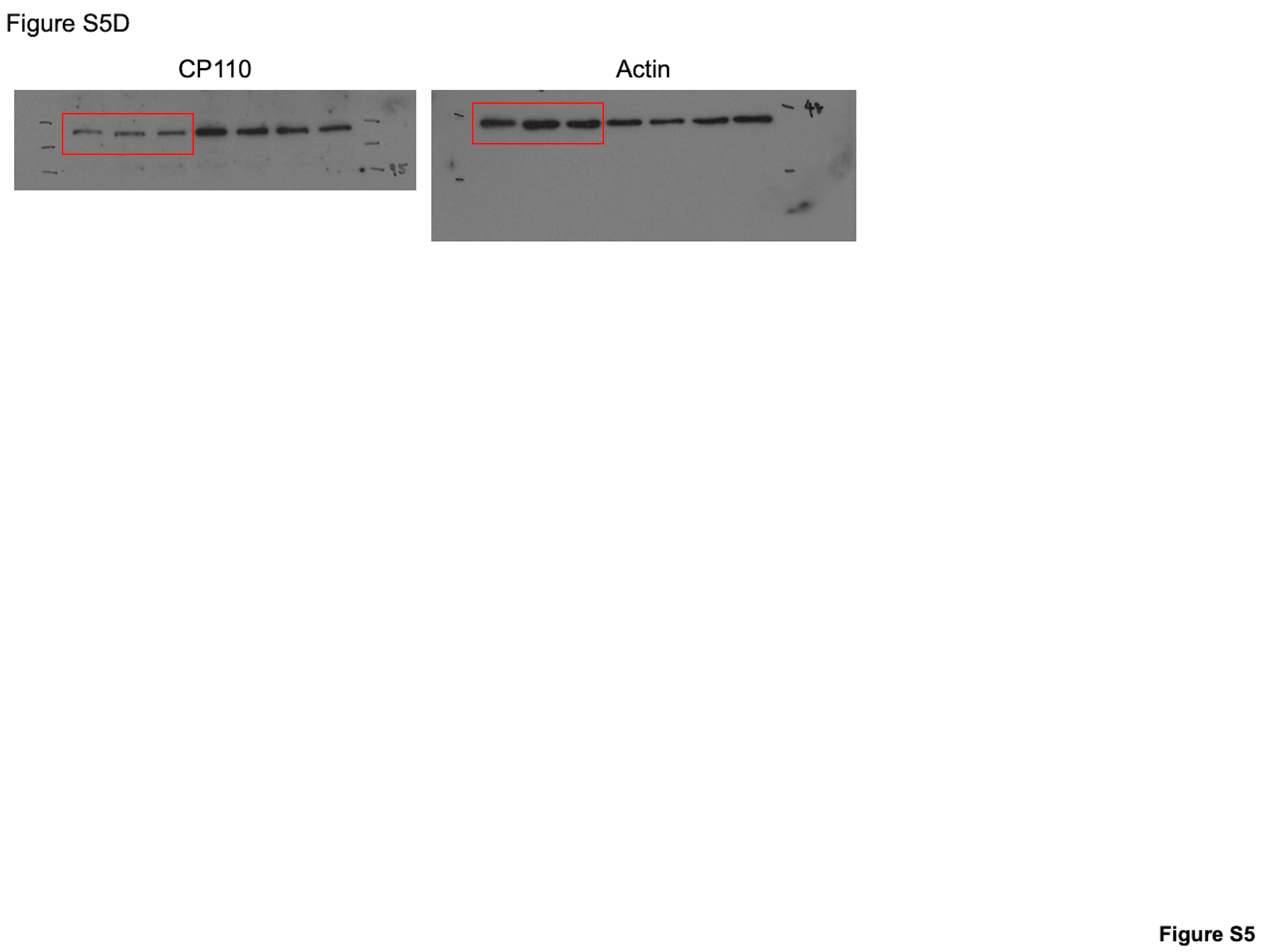

Supplement: Supplementary file 3 [file LSA-2022-01470_SdataFS5.tif]

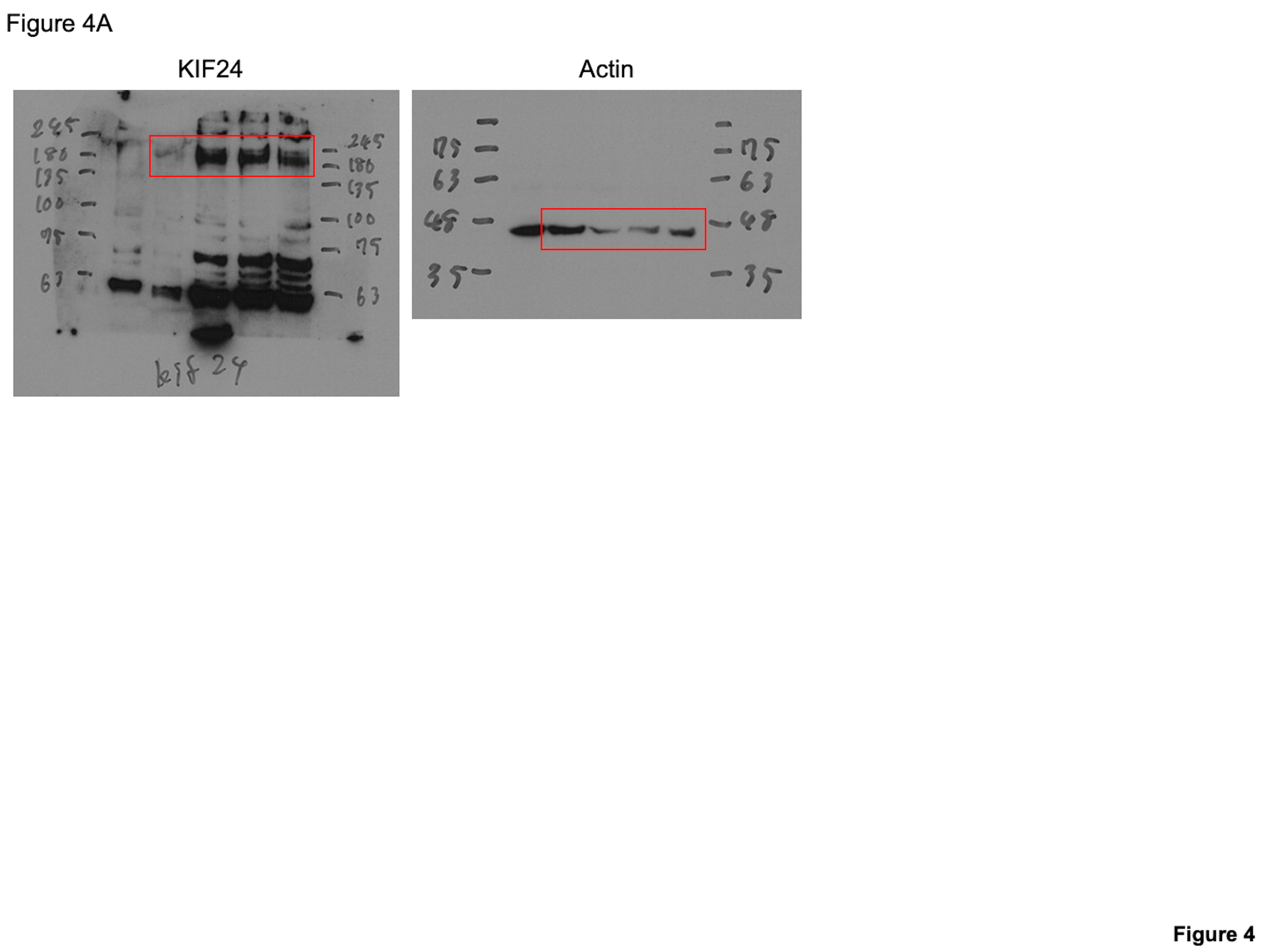

Supplement: Supplementary file 4 [file LSA-2022-01470_SdataF4.tif]
